# SOD3 Decreases Ischemic Injury Derived Apoptosis through Phosphorylation of Erk1/2, Akt, and FoxO3a

**DOI:** 10.1371/journal.pone.0024456

**Published:** 2011-08-31

**Authors:** Lilja E. Laatikainen, Mariarosaria Incoronato, Maria Domenica Castellone, Juha P. Laurila, Massimo Santoro, Mikko O. Laukkanen

**Affiliations:** 1 University of Turku, Medicity Research Laboratory, Turku, Finland; 2 Fondazione IRCCS SDN, Naples, Italy; 3 Institute of Experimental Endocrinology and Oncology (CNR), c/o Department of Biology and Cellular and Molecular Pathology, University of Naples Federico II, Naples, Italy; The University of Texas at Austin, United States of America

## Abstract

**Background:**

Extracellular superoxide dismutase (SOD3), which dismutates superoxide anion to hydrogen peroxide, has been shown to reduce the free radical stress derived apoptosis in tissue injuries. Since both superoxide anion and hydrogen peroxide have a marked impact on signal transduction pathways and could potentially explain a number of apoptosis and survival -related phenomena in different pathological conditions, we clarified the impact of SOD3 on Akt and Erk1/2 cell survival pathways in rat hind limb injury model.

**Methodology and Principal Findings:**

Based on our data, the hind limb ischemic rats treated with virally delivered *sod3* have milder injury and less apoptosis than control animals that could be due to parallel activation of pro-proliferative and anti-apoptotic Erk1/2 and Akt pathways. The common downstream factor of both signaling pathways, the apoptosis related forkhead box protein O3a (FoxO3a), was phosphorylated and translocated to the cytoplasm in *sod3* treated tissues and cell line. Additionally, we obtained increased mRNA production of *elk-1, ets-1, and* microRNA 21 (miR-21), whereas synthesis of *bim* mRNA was decreased in *sod3* overexpressing tissues. We further showed that overexpression of *sod3* modulated redox related gene expression by downregulating *nox2* and *inos* when compared to injured control animals.

**Conclusions and Significance:**

The study shows the complexity of SOD3-derived effects on tissue injury recovery that are not limited to the reduction of superoxide anion caused cellular stress but highlights the impact of SOD3 related signal transduction on tissue functions and suggests an important role for SOD3 in attenuating cell stress effects in different pathological conditions.

## Introduction

Tissue ischemia induces rapid generation of reactive oxygen species (ROS) including superoxide (O_2_^−^•), hydrogen peroxide (H_2_O_2_), and their derivatives, which along with acute lack of nutrient supply and disturbed cellular respiration cause severe damage to tissues. Extracellular superoxide dismutase (SOD3) is an antioxidative enzyme that converts superoxide into hydrogen peroxide thereby reducing oxidative cell stress [1], [2]. The enzyme is secreted to extracellular space where it reversibly binds to cell membrane at lipid rafts and therefore has a local impact on inactivation of phosphotyrosine phosphatases PTP1B and DEP1 [3]. In prolonged disease conditions, such as coronary artery disease, *sod3* expression is decreased in a time-dependent manner [4]–[6] suggesting that the lack of the enzyme could deteriorate the condition. This is further supported by the data showing that *sod3* overexpression has beneficial effects on the healing of cardiovascular injuries [7]–[10]. We have recently shown SOD3 to have a pro-proliferative effect in ischemic skeletal muscle, which is caused by SOD3-derived activation of the Ras-Erk1/2 mitogenic pathway and consequent increased growth factor expression [8] that partially elucidate the SOD3-mediated survival effect. Since the increased proliferation alone may not adequately explain the therapeutic effects caused by SOD3 and, more importantly, since previous studies have shown significantly decreased apoptosis after *sod3* overexpression [7], [9], we focused in the present work on the mechanisms of SOD3-mediated reduced apoptosis in cardiovascular ischemia.

Based on our data, *sod3* overexpression caused activation of Erk1/2 and Akt pathways involving cytoplasmic entry of FoxO3, increased miR-21 production, and decreased BCL-2 interacting mediator of cell death (*bim*) mRNA synthesis. The study suggests an important role for SOD3 in regulation of cellular signaling networks and a central impact on reduced injury development and apoptosis.

## Materials and Methods

### Rat hind limb ischemia model

Acute ischemic hind limb injury was induced in male Fischer 344 rats (5–6 weeks, 86–115g) by surgical ligation of distal femoral artery, lateral circumflex femoral artery and proximal femoral artery. The procedure was performed under anesthesia by single intraperitoneal injection of fentanyl fluanisone (370 µg/100 g; Janssen Pharmaceutica, Beerse, Belgium), and midazolame (180 µg/100 g; Roche, Basel, Switzerland). Adequacy of the anesthesia was determined based on the reactions of the animal during the surgery. Animals were recovering from the anesthesia in a separate pre-warmed environment. Immediately after ligation adenoviral vectors carrying rabbit *sod3* or *Lac*Z control genes, both 0.5×10^9^ pfu in 50 µl PBS, were injected at 5 sites into the hind limb muscles before suturing the wound. Uninjured muscle was used as a control. The animals were followed for 3, 7 and 10 days (4 animals in normal control, LacZ and SOD3 groups each). All animal procedures were approved by the Southern Finland Regional Experimental Animal Committee (License STH350A), and done according to the European Commission and University of Turku guidelines.

### Immunohistochemistry

The thigh muscles were cut crosswise, snap frozen in 2-methylbutane and embedded in Tissue-Tek Optimal Cutting Temperature compound (Sakura Finetek, Torrance, CA, USA). Ten micrometer cryosections were fixed in acetone and stained with hematoxylin/eosin (Sigma, St. Louis, MI, USA) according to the standard protocol, and photographed digitally with Zeiss Axiovert 200 M microscope and the AxioVision program (Carl Zeiss, Oberkochen, Germany). The injured area as percentage of the whole section was determined from 7 sections per group independently by three investigators using inflammatory cell invasion, increased connective tissue formation, and fragmentation of the muscle fibers as criteria for injury analysis. The injured muscle tissue area was calculated by determining the area (%) of the tissue section containing inflammatory cells or morphological damages as compared to inflammatory cell free tissue or tissue that did not show muscle fiber fragmentation of connective tissue. The final injured region was calculated from triplicate analyses (the analysis of three investigators).

### Western blot analysis

Pooled rat muscle tissue from each group was homogenized with a Retsch MM400 mixer mill using metal beads (Retsch GmbH, Haan, Germany) in lysis buffer (50 mmol/l HEPES pH 7.5, 150 mmol/l NaCl, 10% glycerol, 1% Triton X-100, 1 mmol/l MgCl, 10 mmol/l NaF, 10 mmol/l sodium pyrophosphate, 1 mmol/l Na3VO4, 10 mmol/l approtinin, 10 µg/ml leupeptin) (Sigma). Antibodies for cleaved caspase-3 (Asp175), p-Akt (Ser473), Akt, p-Erk1/2 (Trh202/Tyr204), Erk1/2, p-FoxO3a (Ser318/321), p-FoxO3a (Thr32), tubulin (Cell signaling, Danvers, MA, USA), and SP-1 (Santa Cruz, Santa Cruz CA, USA) were used to detect the designated proteins from the blotted samples.

### Cell culture and cell fractionation

NIH 3T3 cells (ATCC, Teddington, UK) were grown in DMEM 10% CS (Sigma) with penicillin-streptomycin (Sigma). Empty pcDNA3 control vector or human *SOD3* cDNA (a kind gift from professor Stefan L. Marklund, University of Umeå, Sweden) subcloned into the pcDNA3 plasmid, were stably transfected using Fugene 6 (Roche, Mannheim, Germany). The cells were grown in the presence of geneticin (Sigma) and prepared for cell fractionation. Nuclear and cytoplasmic fractions of the cells were isolated using NE-PER Cell Fractionation kit (Thermo Scientific, Waltham, MA, USA). The cells were lysed with the NE-PER nuclear and cytoplasmic extraction reagents. The fractionated proteins were loaded on SDS gels according to the standard procedures.

### Real-time quantitative PCR

Total RNA was extracted from pooled muscle samples of each animal group with Tri-reagent (Sigma). Complementary DNA synthesis was done with Revert-Aid M-MuLV (Fermentas, Burlington, Canada) and the quantitative PCR with SYBR Green master mix reagent (Applied Biosystems, Foster City, CA, USA). Primers were: rat *beta-actin* forward 5′-TCGTGCGTGACATTAAGGAG-3′ and reverse 5′-GTCAGGCAGCTCGTAGCTCT-3′; endogenous rat *sod3* forward 5′-GAC CTG GAG ATC TGG ATG GA-3′ and reverse 5′-GTG GTT GGA GGT GTT CTG CT-3′; exogenous rabbit *sod3* forward 5′-GTT GCG TGA GCG GAA AGA TG-3′ and reverse GTG AGC GCC TGC CAG ATC TC; *nox2* forward 5′-TTG TTG CAG GAG TGC TCA TC-3′ and reverse 5′-CTG CCA GCA GGT AGA TCA CA-3′; *inos* forward 5′-GGT GCA GAA GCA CAA AGT CA-3′ and reverse 5′-GAA CTG GGG GAA ACC ATT TT-3′; *elk-1* forward 5′-AGC GGC CAG AAG TTT GTC TA-3′ and reverse 5′-CTG TCA TTC CTG CAC CCT TT-3′; *ets-1* forward 5′-GAA ATG ATG TCC CAG GCA CT-3′ and reverse 5′-CTT TAC CCA GGG CAC ACA GT-3′; *bim* forward 5′-ATC TCA GTG CAA TGG CTT CCA-3′ and reverse 5′-GCT CCT GTG CGA TCC GTA TC-3′; *miR21* and RNU5 miScript Primer Assays (Qiagen, Hilden, Germany) were used to study the amplification and to normalize the miR-21 expression.

### Statistical analyses

The experiments were repeated at least three times. All results are expressed as mean ±SD. The p-values (* = p<0.05, ** = p<0.01, *** = p<0.001) were determined by one-way Anova with Tukey-Kramer multiple comparison post-analysis test.

## Results

### SOD3 attenuates ischemic injury

Previously SOD3 has been shown to have growth regulatory [8], anti-inflammatory [11], anti-oxidative [2], and anti-apoptotic [7], [9] characteristics. Since the latter is not well characterized, in the present study we focused on *sod3* overexpression-mediated cellular signaling events leading to decreased apoptotic signaling in ischemic tissue injury.

**Figure 1:**
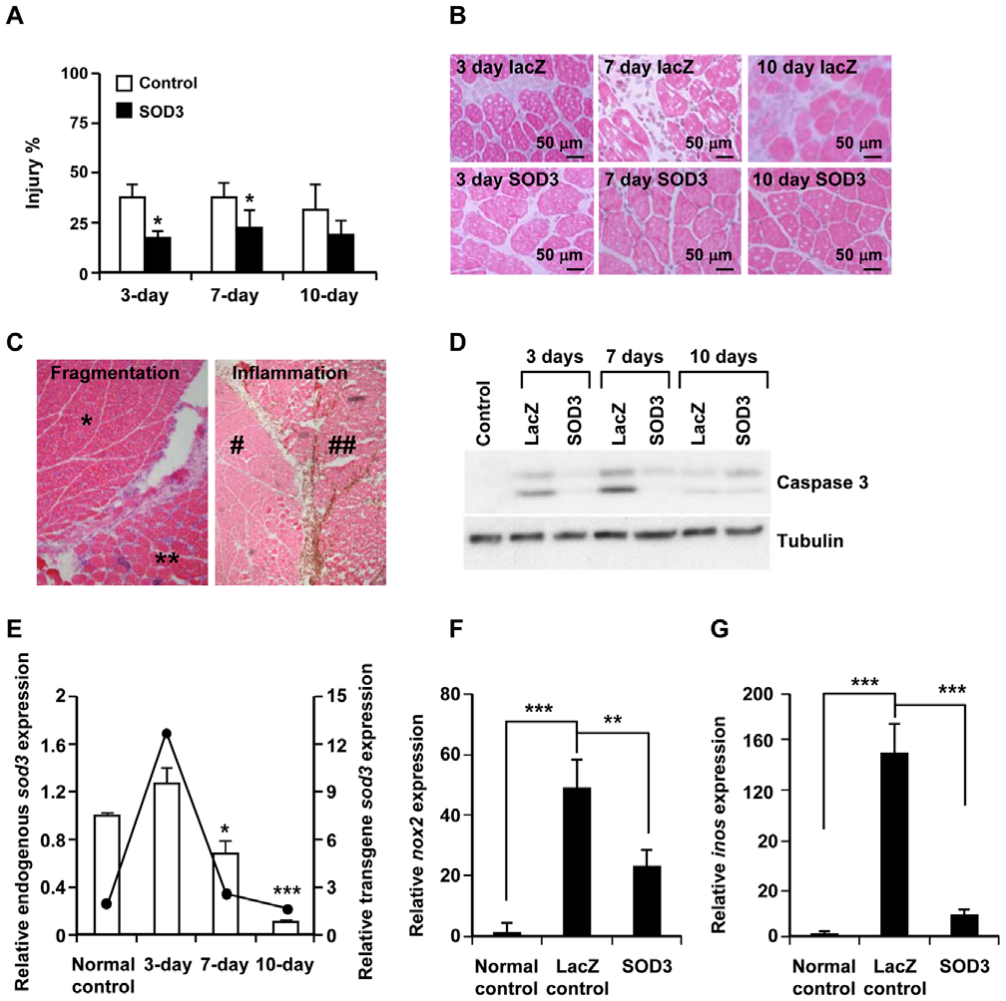
SOD3 overexpression affects tissue injury development and modifies redox enzyme expression. (A) *Sod3* transduced ischemic muscle tissues had significantly lower injury score at 3-day (p<0.05 (*)) and at 7-day (p<0.05 (*)) time points. White bars refer to *lacz* control animals and black bars to *sod3* transfected rats. (B) Corresponding hematoxylin/eosin staining showed increased fibrosis in LacZ control animals in different time points as compared to SOD3 treated muscles. (C) The edges of the injured region were clearly visible. The left panel shows hematoxylin/eosin staining of normal uninjured (*) tissue region and injured fragmented (**) muscle section. In the right panel there is an inflammatory cell free region (#) and heavily positive CD68 inflammation muscle area (##). Since the fragmented and inflammatory cell positive regions were partially different the injured muscle tissue area was analyzed using both parameters separately. (D) Western blot with cleaved caspase-3 (17 kDa and 19 kDa) antibody indicated increased apoptosis at 3-day and 7-day time points in LacZ control animals as compared to *sod3* treated animals. (E) Rat endogenous *sod3* mRNA expression (open bars) was downregulated at 7-day and 10-day time points (p>0.05 (*) and p<0.001 (***), respectively). The overexpression of transgene *sod3* (line) was highest 3 days after the gene transfer and reduced to background levels by the day 10. The left Y-axis refers to relative endogenous *sod3* and the right Y-axis relative transgene *sod3* expression. (F) Ischemic injury caused significantly (p<0.001) increased *nox2* mRNA expression as compared to normal uninjured control animals. *Sod3* overexpression attenuated the increase causing a significant (p<0.01 (**)) reduction of *nox2* expression as compared to 3-day LacZ control animals. (G) Injury-related increased (p<0.001 (***)) *inos* expression was significantly (p<0.001 (***) decreased by *sod3* expressing tissues.

To justify that the *in vivo* model used in the study is applicable for the present aims we first determined the relative sizes of injured tissue areas from the histological cryo sections. We observed significantly smaller injury regions in *sod3*-treated animals as compared to control rats on 3-day (53% lower value, p<0.05) and on 7-day (40% lower value, p<0.05) time points (Figure 1A-C). To further characterize the effect of SOD3 and to verify whether the enzyme has an impact on modulating apoptosis in our model, we performed a cleaved caspase-3 Western blot that showed increased apoptotic activity in *lacz* control animals as compared to *sod3*-treated rats at 3-day and at 7-day time points (Figure 1D).

Since it has been reported that the cardiovascular damages are characterized by reduced *sod3* expression [4]–[6] we analyzed the expression of endogenous rat *sod3* mRNA in ischemic skeletal muscle from the control animals at different time points. At 3-day time point there was an initial increase in *sod3* mRNA synthesis that was followed by 2-fold (p<0.05) reduction at 7-day time point and 10-fold reduction (p<0.001) at 10-day time point as compared to normal uninjured muscle (Figure 1E). The down-regulation of endogenous *sod3* expression in injured tissues was paralleled by increased levels of pro-inflammatory molecules, such as *NADPH oxidase 2* (*nox2*) and inducible nitric oxide synthase (*inos*) that, as previously shown [9], [12], [13] can be modulated by *sod3* overexpression. The injury-related marked increase in *nox2* and *inos* mRNA production (Figure 1F–G) could be due to macrophage infiltration into the ischemic injury region [14]. However, the expression of both enzymes was significantly (p<0.01 and p<0.001, respectively) attenuated by *sod3* overexpression as compared to *lacz* control muscles. These observations from animal tissues indicate that SOD3 overexpression has a significant impact on tissue injury related gene expression.

### Erk1/2 pathway is the principal pathway in SOD3-mediated tissue recovery

**Figure 2:**
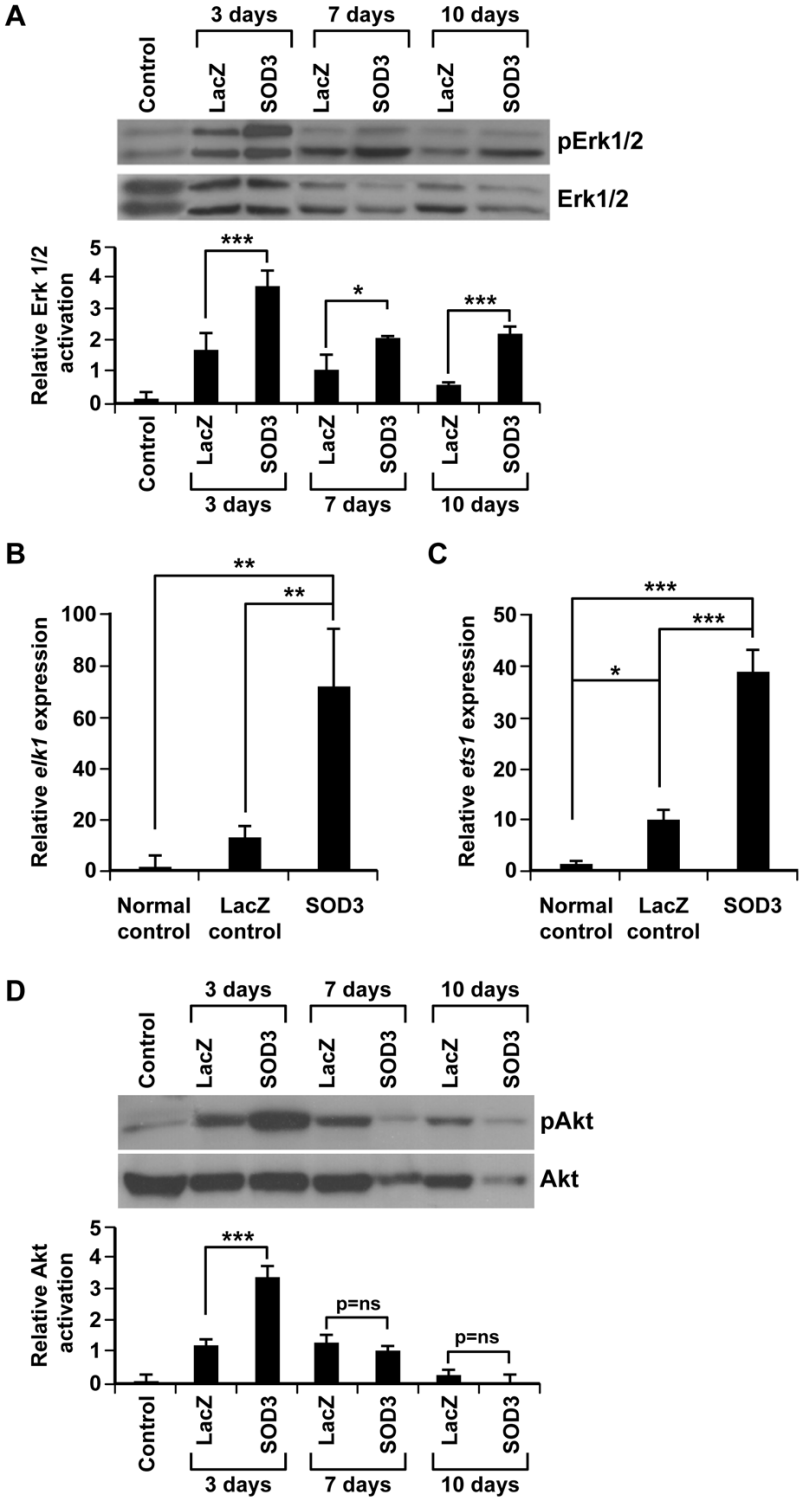
The effect of SOD3 on Erk1/2 signaling pathway. (A) Western blot analysis showed increased Erk1/2 phosphorylation in *sod3* transduced muscles at 3-day, 7-day, and 10-day time points (p<0.001 (***), p<0.05 (*), and p<0.001 (***), respectively) as compared to corresponding *lacz* controls. The samples were normalized with total Erk1/2 antibody. Erk1/2 activation was supported by significant up-regulation of its downstream transcription factors (B) *elk-1* (p<0.01 (**)) and (C) *ets-1* (p<0.001 (***)), measured by mRNA synthesis in *sod3* animals as compared to LacZ control tissues. The injury itself moderately increased production of both molecules as compared to normal uninjured tissue control. However, the significant difference between normal control tissue and LacZ control (p<0.05 (*)) was achieved only in *ets-1* expression. (D) *Sod3* transduced muscles have increased levels of Akt phosphorylation only at 3-day time point (p<0.001 (***)). The samples were normalized with total Akt antibody.

To explain the reduced injury development and reduced apoptosis in our hind limb injury model we next studied the cell survival signaling pathways that become activated in *sod3*-treated tissues. Since we have previously proved that *sod3* is able to modulate the activation of Ras-Erk1/2 signaling pathway [8], which is a major regulator of cell growth and survival, we verified the level of Erk1/2 phosphorylation in injured control and *sod3* treated animals as compared to uninjured tissues. Figure 2A shows that the injury itself is causing an increase of phospho-Erk signaling, and that this effect is significantly up-regulated by *Sod3* treatment at 3-day (p<0.001), 7-day (p<0.05), and 10-day (p<0.001) time points. We further investigated the effect of SOD3 in modulating the activity of Erk1/2 pathway by monitoring its downstream target transcription factors Elk-1 and Ets-1 [15], [16]. Both *elk-1* and *ets-1* expressions were significantly (p<0.01 and p<0.001, respectively) increased in *sod3* animals as compared to LacZ controls (Figure 2B–C). Interestingly, the injury itself had a minor effect on the synthesis of these two transcription factors, suggesting that *sod3* overexpression plays an important role in the prolonged activation of mitogenic signal transduction routes controlling cell proliferation and tissue recovery. We then focused on the effect of SOD3 on the regulation of PI3K/Akt anti-apoptotic pathway to determine the reduced caspase-3 cleavage observed in *sod3* treated animals by investigating phosphorylation of Akt in injured tissues. Our results showed that *sod3* stimulated phosphorylation of Akt was visible only at early time point (3 days after the injury) (Figure 2D), whereas the simultaneous effect on Erk1/2 phosphorylation was long-lasting (up to 10 days) (Figure 2A).

### SOD3-mediated anti-apoptotic signaling

**Figure 3:**
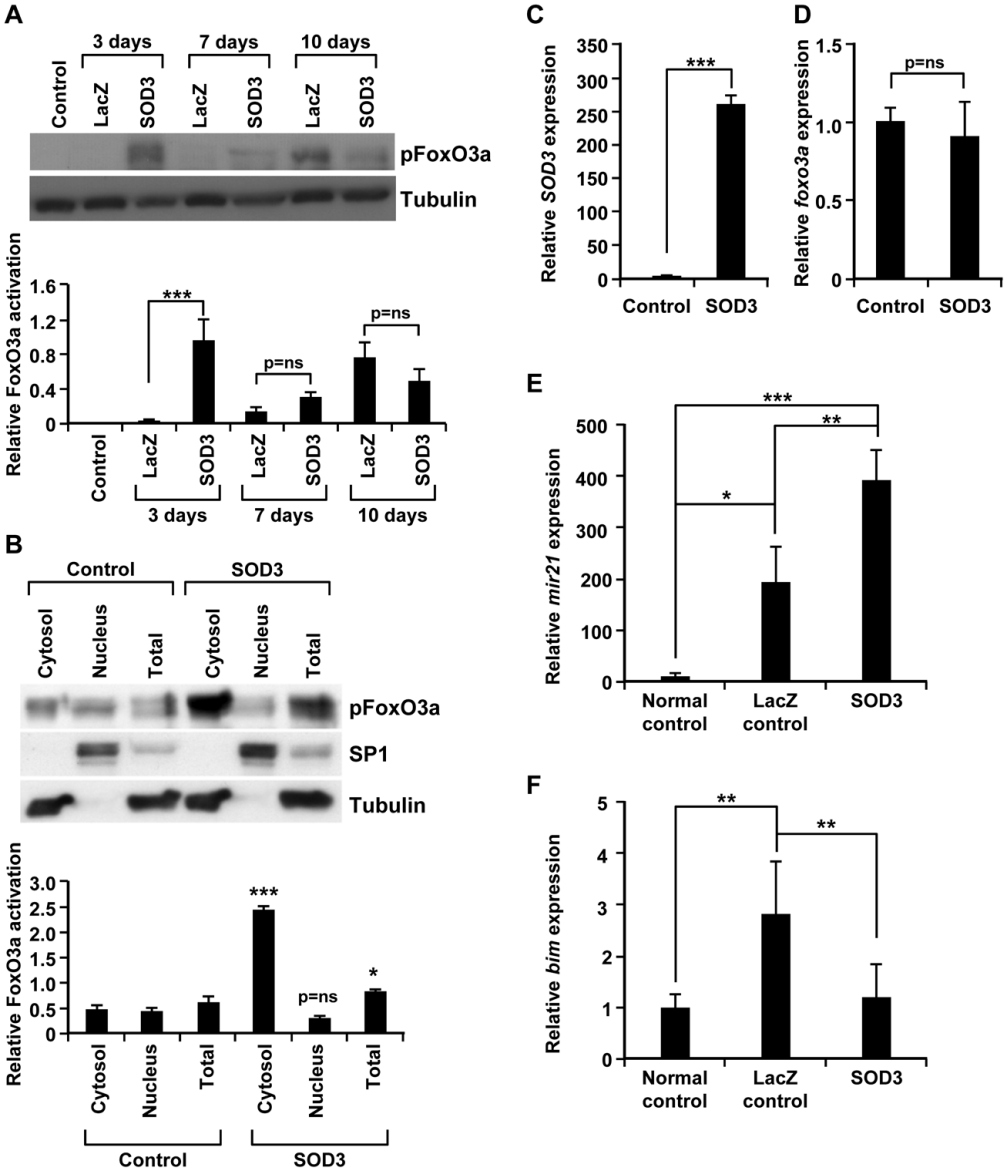
SOD3-mediated anti-apoptotic signaling. (A) The phosphorylation of FoxO3a (Thr32) in *sod3* treated muscle tissues was strongly up-regulated at 3-day time point, had non-significant tendency at 7-day time point and showed no difference to LacZ control tissues at 10-day time point. The blot was normalized with tubulin antibody. (B) *In vitro* cell fractionation assay showed increased phosphorylation of FoxO3a in *SOD3* stably transfected NIH 3T3 cells. The amount of phosphorylated protein was significantly (p<0.05 (*)) higher in the total cell extract and was enriched in the cytoplasmic fractions (p<0.001 (***)) of cells transfected with *SOD3* when compared to control cells. Normalization was performed by using α-SP1 antibody for the nuclear fractions and α-tubulin antibody for the cytoplasmic fraction. For statistical analysis cytosol, nucleus, and total protein compartments were compared between control and *SOD3* samples. (C–D) To determine the effect of SOD3 on the amount of total FoxO3a, we measured in NIH3T3 cells stably transfected with *SOD3* (panel C) the mRNA synthesis of *foxo3a* (panel D) that showed no difference to control cells. (E) To verify whether FoxO3a targets were also regulated by SOD3 we then performed expression analysis for *mir-21* in rat tissues showing significantly (p<0.05 (*)) increased miRNA synthesis caused by the ischemic injury that was further induced by *sod3* overexpression (p<0.01 (**)) as compared to LacZ control animals). (F) Similarly, also the expression of *bim*, which is another target of FoxO3a, was found to be modulated by SOD3, as its expression was significantly stimulated (p<0.01 (**)) due to ischemia and then decreased by *sod3* overexpression (p<0.01 (**)).

It has been shown that co-operative action of Akt and Ras-Erk1/2 signaling cascades mediate anti-apoptotic and pro-proliferative signals by e.g. causing FoxO3a phosphorylation, inactivation and consequent cytoplasmic entry [17], [18] that in ischemic tissues has been reported to result in reduction of transcriptional effect on FoxO3a target genes [19]. We therefore investigated whether SOD3, by modulating Erk1/2 and Akt activation, could also affect FoxO3a phoshorylation status. Based on our current data SOD3 causes increased FoxO3a phosphorylation at 3-day time point (Figure 3A). Since phosphorylation determines the nuclear/cytoplasmic location of FoxO3a activity, we further characterized the SOD3-derived effect on FoxO3a localization in NIH 3T3 cells. The cells were stably transfected with *SOD3* or control eukaryotic expression vector, fractionated to separate nuclear and cytoplasmic compartments, and analyzed by Western blotting. According to our results *sod3* transfected cells had markedly (p<0.05) more phosphorylated total FoxO3a, and interestingly the amount of phosphorylated protein was significantly (p<0.001) enriched in the cytosolic compartment as compared to control vector transfected cells (Figure 3B). To determine the effect of SOD3 on the total amount of FoxO3a, we then performed a real time PCR analysis and found that *SOD3* expressing cells (Figure 3C) had similar levels of *foxo3a* mRNA as compared to control cells (Figure 3D), suggesting that SOD3 is mainly influencing the phosphorylation levels and consequent cytoplasmic entry of FoxO3a, but not the total protein amount.

Since it was recently shown that in the nucleus FoxO3a suppresses the anti-apoptotic miR-21 transcription [20], [21] we next analyzed the microRNA (miRNA) expression. Based on the quantitative real time-PCR data, the expression of *mir-21* in *sod3* overexpressing ischemic tissues at 3-day time point was significantly (p<0.001) increased as compared to uninjured control muscles. Even though the injury itself had an activating effect on miRNA expression (p<0.05) *sod3* was able to significantly (p<0.01) further increase it as compared to LacZ injured control tissues (Figure 3E). To characterize another FoxO3a downstream target we then checked the mRNA expression of pro-apoptotic *bim*, which is known to be downregulated by coordinated action of Akt and Erk1/2 [22]–[24]. As shown in figure 3F, the injury-related increased (p<0.01) *bim* mRNA expression was significantly (p<0.01) decreased in *sod3* tissues thereby indicating that the SOD3-derived activation of Akt in coordination with Erk1/2 signal transduction routes may represent a key mechanism to reduce the apoptotic response in tissues.

## Discussion

The skeletal muscle ischemia induces production of ROS by the disrupted metabolism and by the infiltrating inflammatory cells, such as macrophages. The physiological function of SOD3 is to convert superoxide anion to hydrogen peroxide on the extracellular side of the cell membrane [1], [2] relieving the free radical superoxide anion derived damages in the tissue environment. However, this reaction leading to reduced oxidative stress does not explain *per se* the decreased apoptosis seen in SOD3 overexpressing tissues. Therefore, our purpose in the current work was to clarify the PI3K-Akt and Erk1/2 response caused by SOD3 in apoptosis model.

Based on the current data the significantly increased *nox2* and *inos* mRNA expressions together with decreased *sod3* expression at later time points (Figure 1E–G) suggest a marked imbalance in redox enzyme expression levels in developing tissue injury. Previously, we have shown that *sod3* gene transfer to ischemic hind limb injury is able to increase the active SOD3 concentration in the tissue by 2-fold [8], which together with the remarkably long half-life of the enzyme in the muscle tissue, up to 100 hours [25], would be able correct the decrease of the endogenous enzyme expression causing the therapeutic response. The current analysis of the effect of *sod3* overexpression on the apoptosis (Figure 1D) was in line with previous reports demonstrating the anti-apoptotic role of the enzyme [7], [9] further suggesting SOD3-derived response on cell survival signaling.

Among the anti-apoptotic and pro-survival pathways that are activated after ischemic injuries, the PI3K-Akt and Erk1/2 routes are considered to be the most important. The numerous substrates of these kinases include several apoptosis-related factors such as Foxo3a and caspases, which are affected by Akt and/or Erk phosphorylation [17], [18], [26]–[28]. The interplay between SOD3 and Akt has been confirmed previously in an *in vitro* experiment in which *sod3* transfection increased phospho-Akt levels in cells but not at late time point in rat tissues [8]. In the present work, we found notably increased level of *sod3* promoted Akt phosphorylation at early phase of the injury that, however, was lost at later phase unlike Erk1/2, suggesting milder SOD3-related stimulation to PI3K-Akt than Erk1/2 signaling. To further strengthen the role of Erk1/2 in cell survival we showed upregulation of *ets-1* and *elk-1* transcription factors in *sod3* treated animals as compared to controls (Figure 2B–C), which let us to speculate that the earlier robust Erk1/2 activation induced by SOD3 continuously boosts the *ets-1* and *elk-1* expression, lifting it to a significantly higher level than in the control animals.

One of the direct Erk1/2 and Akt target proteins, the ROS responsive transcription factor FoxO3a, regulates the expression of many cell cycle arrest- and apoptosis-related genes [29]–[32] and is also known to protect normal quiescent cells from oxidative stress by regulating several antioxidant genes e.g. peroxiredoxins, glutathione peroxidases, and superoxide dismutases including SOD3 [33]–[35]. We discovered increased FoxO3a phosphorylation in *sod3* treated animals at day 3 after the ischemic injury, which was then decreased to the level of the control animals (Figure 3A). The effect of *sod3* overexpression on FoxO3a phosphorylation and cytoplasmic entry was confirmed in *sod3* stable NIH3T3 cell line that demonstrated increased phosphorylation of the transcription factor while the total amount of *foxo3a* remained unaffected (Figure 3B–D).

Recently, FoxO3a was found to negatively regulate an anti-apoptotic microRNA, *mir-21* by binding the *mir-21* promoter to suppress its expression [21]. In line with this, we demonstrated increased *mir-21* expression *in vivo* in *sod3*-transduced tissues (Figure 3E). Next, to confirm that *sod3* is affecting FoxO3a function we monitored the inhibition of *bim*, another common downstream target of Akt and Erk1/2 pathways. BH3-only proteins, such as BIM, are cell death initiators that are activated in cellular stress conditions including response to DNA damages, decreased metabolism, growth factor withdrawal, and hypoxia. Based on our results the significantly (p<0.01) decreased expression of *bim* mRNA production correlated with *sod3* overexpression in muscles suggesting that SOD3-derived increased FoxO3a phosphorylation at 3-day time point might be able to attenuate the initiation of the apoptotic process by BH3-only protein BIM (Figure 3F).

**Figure 4:**
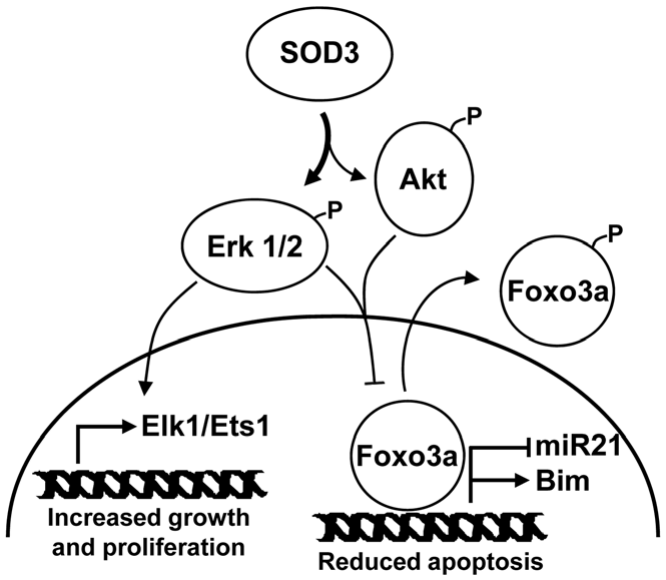
Schematic representation of the SOD3 action in promoting the cell survival in injured tissues. Temporal simultaneous activation of Erk1/2 and Akt leads to phosphorylation and consequent cytoplasmic translocation of FoxO3a, which then increases miR-21 production and downregulates the *bim* mRNA expression. Erk1/2 activation further stimulates the cell survival signaling by increasing cell proliferation related transcription factor production.

In summary, we have elucidated the signaling events by which the extracellular *sod3* promotes cell survival and tissue recovery in skeletal muscle ischemia model (Figure 4). The overexpression of *sod3* correlated with simultaneous activation of Akt and Erk1/2, consequent FoxO3a cytoplasmic entry, anti-apoptotic *mir-21* upregulation and pro-apoptotic *bim* mRNA downregulation. On the tissue level, *sod3* overexpression attenuated the injury development and decreased apoptosis. Therefore, the present data support our previous findings connecting the Erk1/2 mitogenic signaling cascade and downstream effectors to *sod3* expression and cell survival effects.
